# Genome Sequence of *Roseovarius mucosus* Strain SMR3, Isolated from a Culture of the Diatom *Skeletonema marinoi*

**DOI:** 10.1128/genomeA.00394-17

**Published:** 2017-06-01

**Authors:** Mats Töpel, Matthew I. M. Pinder, Oskar N. Johansson, Olga Kourtchenko, Anna Godhe, Adrian K. Clarke

**Affiliations:** a Department of Marine Sciences, University of Gothenburg, Göteborg, Sweden; b Gothenburg Global Biodiversity Centre, Göteborg, Sweden; c Department of Biological and Environmental Sciences, University of Gothenburg, Göteborg, Sweden

## Abstract

We present the genome of *Roseovarius mucosus* strain SMR3, a marine bacterium isolated from the diatom *Skeletonema marinoi* strain RO5AC sampled from top layer sediments at 14 m depth. Its 4,381,426 bp genome consists of a circular chromosome and two circular plasmids and contains 4,178 coding sequences (CDSs).

## GENOME ANNOUNCEMENT

*Roseovarius mucosus* strain SMR3 was isolated from a culture of the chain-forming diatom *Skeletonema marinoi*. The diatom strain (RO5AC) was established from a germinated resting cell embedded in sediment. Sediment was collected with a box corer in Öresund, Sweden (55°59.16′ N, 12°44.02′ E), in May 2010. The genome was sequenced on one single-molecule real-time (SMRT) cell using the PacBio RSII system (Pacific Biosciences, Menlo Park, CA, USA), which produced 83,546 uncorrected reads totaling 756.4 Mbp. Reads were assembled using Falcon version 1.7.5 (https://github.com/PacificBiosciences/FALCON/ [1]) with a seed read length of 7,000 bp, and circularization of contigs was confirmed by joining the corresponding ends and realigning the reads using the RS_Resequencing.1 protocol on SMRT Portal version 2.3.0 (Pacific Biosciences) (2). The resulting assembly contained three circular contigs totaling 4,381,426 bp, with average read coverage of 144.99×.

The chromosome is 4,170,996 bp long with a G+C content of 60.9%, plasmid pSMR3-1 is 180,135 bp (G+C 60.1%) and plasmid pSMR3-2 is 30,295 bp (G+C 58.3%). The two identical 16S rRNA sequences found in the chromosome have 99.8% identity with that of *R. mucosus* DSM 17069^T^ (accession no. AONH01000000). Sequences identical to the SMR3 16S sequences were also found at three locations in *Roseovarius* sp. TM1035 (accession no. NZ_ABCL00000000). A phylotaxonomic analysis using PhyloPhlAn version 0.99 (3), comparing the SMR3 strain to all whole-genome sequenced strains in the family *Rhodobacteraceae* available at the NCBI RefSeq ftp site (ftp://ftp.ncbi.nlm.nih.gov/genomes/refseq/bacteria/), showed it to be sister to *Roseovarius* sp. TM1035, and together with *Roseovarius mucosus* strain DSM 17069^T^ forming a clade with 100% bootstrap support. Annotation was performed using Prokka version 1.12beta (4), which predicted 4,178 coding sequences (CDSs) (3,542 with a functional prediction and 636 hypothetical proteins), 3 pseudogenes, 46 tRNAs, 6 rRNAs, 5 noncoding RNAs (ncRNAs), and 1 tmRNA.

*Roseovarius mucosus* strain SMR3 contains two phosphatidylcholine synthase genes (ROSMUCSMR3_00993 and ROSMUCSMR3_02532), and both an acyl-homoserine-lactone synthase and *luxR* gene (ROSMUCSMR3_00613 and ROSMUCSMR3_00149, respectively; both involved in quorum sensing), all of which are suggestive of a symbiotic or pathogenic relationship with a eukaryote (reviewed in references 5 and 6). There is also evidence of interaction in the form of horizontal gene transfer between *R. mucosus* strain SMR3 and other bacteria within the *S. marinoi* microbiome. The pSMR3-2 plasmid shares 100% sequence identity, over the full length of the plasmid, with interrupted regions of a *Loktanella* sp. plasmid found in the same *S. marinoi* culture. These interruptions can primarily be accounted for by transposase genes on the *Loktanella* sp. plasmid.

### Accession number(s).

This whole-genome project has been deposited in GenBank under the accession numbers CP020474, CP020475, and CP020476, as part of BioProject No. PRJNA380207.
